# Supplementary material for: Identification of photoperiod-induced specific miRNAs in the adrenal glands of Sunite sheep (Ovis aries)
Source: Front Vet Sci. 2022 Jul 22;9:888207. doi: 10.3389/fvets.2022.888207 (PMC9354845; doi:10.3389/fvets.2022.888207)
Supplement: Supplementary file 1 [file Data_Sheet_1.ZIP › Supplementary file/miRDeep2 file.zip!/miRDeep2 file/Novel_Structure/SPLP42A3/Novel_702.pdf]

Provisional ID	:	NC_019461.2_12265
Score total	:	0
Score for star read(s)	:	-1.3
Score for read counts	:	-2.8
Score for mfe	:	2.5
Score for randfold	:	1.6
Score for cons. seed	:	
Total read count	:	6
Mature read count	:	3
Loop read count	:	0
Star read count	:	3

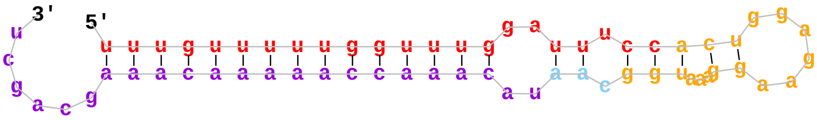

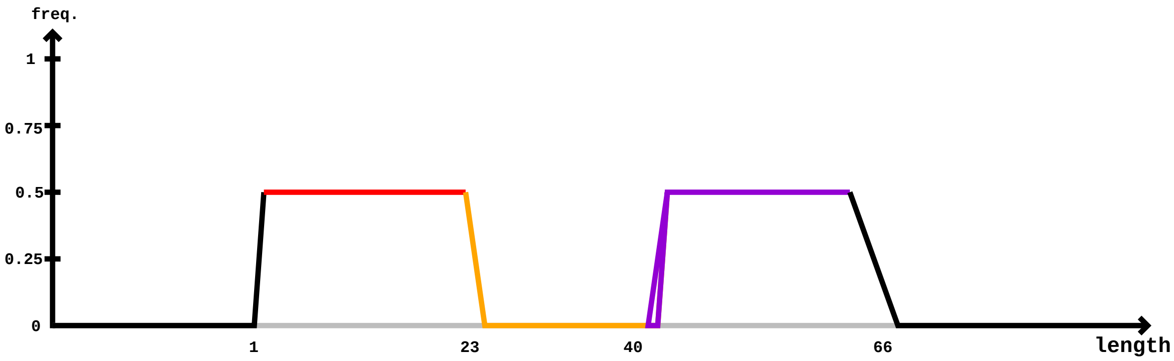

Mature **Star**

```
5'- cgggcgggguuauuagcugcuuuguuuuugguuuggauuuccacuggagaaggaaauggcaauacaaaccaaaaacaaagcagcuaaaauaaccccgccuguuuagaaugau  -3'   obs
    cgggcgggguuauuagcugcuuuguuuuugguuuggauuuccacuggagaaggaaauggcaauacaaaccaaaaacaaagcagcuaaaauaaccccgccuguuuagaaugau         exp
    (((((((((((((((((((((((((((((((((((..((.(((((......))...))).))..))))))))))))))))))))))..)))))))))))))...........   reads    mm    sample
    ....................uuuguuuuugguuuggauuucc......................................................................   3        0     seq
    ..............................................................uacaaaccaaaaacaaagcag.............................   1        0     seq
    ..............................................................uacaaaccaaaaacaaagcagcu...........................   2        0     seq
```
